# p19^Arf^ Exacerbates Cigarette Smoke-Induced Pulmonary Dysfunction

**DOI:** 10.3390/biom10030462

**Published:** 2020-03-17

**Authors:** Ryuta Mikawa, Tadashi Sato, Yohei Suzuki, Hario Baskoro, Koichiro Kawaguchi, Masataka Sugimoto

**Affiliations:** 1 Research Institute, National Center for Geriatrics and Gerontology, Obu, Aichi 474-8511, Japan; mikawa@ncgg.go.jp (R.M.); kk-choju@ncgg.go.jp (K.K.); 2 Department of Respiratory Medicine, Juntendo University Graduate School of Medicine, Tokyo 113-8421, Japan; yohei-s@juntendo.ac.jp (Y.S.); hario@juntendo.ac.jp (H.B.); 3 Department of Molecular Aging Research, Nagoya University Graduate School of Medicine, Nagoya 466-8560, Japan

**Keywords:** p19^Arf^, senescence, senolysis, emphysema, COPD

## Abstract

Senescent cells accumulate in tissues during aging or pathological settings. The semi-genetic or pharmacological targeting of senescent cells revealed that cellular senescence underlies many aspects of the aging-associated phenotype and diseases. We previously reported that cellular senescence contributes to aging- and disease-associated pulmonary dysfunction. We herein report that the elimination of *Arf*-expressing cells ameliorates cigarette smoke-induced lung pathologies in mice. Cigarette smoke induced the expression of *Ink4a* and *Arf* in lung tissue with concomitant increases in lung tissue compliance and alveolar airspace. The elimination of *Arf*-expressing cells prior to cigarette smoke exposure protected against these changes. Furthermore, the administration of cigarette smoke extract lead to pulmonary dysfunction, which was ameliorated by subsequent senescent cell elimination. Collectively, these results suggest that senescent cells are a potential therapeutic target for cigarette smoking-associated lung disease.

## 1. Introduction

Chronic obstructive pulmonary disease (COPD) is a severe and incurable disease with a relatively high prevalence and is currently one of the leading causes of death worldwide [1]. A major component of COPD is pulmonary emphysema, which is characterized by enlarged alveolar airspaces and alveolar wall collapse. Cigarette smoke (CS) is the dominant cause of the disease, and aging also increases the risk of COPD [2]. COPD is associated with the infiltration of inflammatory cells, which is considered to cause the accumulation of proteinases and lead to alveolar destruction [3,4]. The inhibition of macrophage or neutrophil elastase was previously shown to be sufficient to suppress the pathologies of CS-induced emphysema in mice [5,6]. Cellular senescence is also accelerated in emphysema patients [7]. However, the roles of cellular senescence in this disease have yet to be characterized.

Cellular senescence acts as a potent tumor suppression mechanism in mammals by halting the proliferation of cells at risk of malignant transformation [8]. Cellular senescence is mediated by the *Cdkn2a* locus, which encodes two important tumor suppressors, namely, p16^Ink4a^ and p19^Arf^ (p14^ARF^ in humans) [9,10]. These proteins indirectly activate the Rb and p53 tumor suppressors, respectively, thereby inducing and maintaining cell cycle arrest during cellular senescence. In addition to permanent cell cycle arrest, it has become apparent that senescent cells produce a series of cytokines, chemokines and extracellular proteinases, thereby non-cell autonomously affecting the functions of their surrounding non-senescent cells. These are collectively called the senescence-associated secretory phenotype (SASP), and increasing evidence suggests that SASP mediates the deleterious effects of cellular senescence and is involved in a number of pathologies in both humans and mice [11].

Senescent cells accumulate in several tissues during aging [12,13], and are considered to contribute to aging-associated pathologies. Recent studies on senolysis (senescent cell elimination) using semi-genetic and pharmacological approaches revealed that the elimination of senescent cells from tissues ameliorates a number of aging-associated tissue dysfunctions and diseases [11,14] and even extends the lifespan of mice [15]. Cellular senescence is also involved in lung disease, and bleomycin-induced idiopathic pulmonary fibrosis (IPF) was shown to be alleviated by the elimination of senescent cells in INK-ATTAC mice or the administration of the senolytic drugs dasatinib and quercetin [16]; however, the efficacy of these drugs in human IPF patients currently remains unclear [17].

We also established transgenic mice (ARF-DTR mice) that express luciferase and diphtheria toxin (DT) receptors under the control of the *Arf* promoter/enhancer, thereby enabling the detection and ablation of *Arf*-expressing cells by in vivo imaging and DT, respectively [18]. Using ARF-DTR mice, we showed that the elimination of *Arf*-expressing cells restored pulmonary function in aged animals [19]. Furthermore, the pre-elimination of *Arf*-expressing cells by DT protected lung tissue against elastase-induced lung injury [20]. This effect may be mediated by reduced pulmonary inflammation exemplified by an increased number of alveolar macrophages. Similar findings were also obtained using the anti-apoptotic Bcl-2 family protein inhibitor ABT-263, which has been shown to exhibit senolytic activity in mice [21]. Collectively, these findings suggest that the elimination of senescent cells exerts protective effects against elastase-induced pulmonary emphysema. However, it currently remains unclear whether the elimination of senescent cells is also effective in CS-induced pulmonary injury, which is more closely related to common human diseases.

In the present study, we investigated the effects of *Arf*-expressing cells on CS-induced lung pathologies in mice. Chronic CS exposure resulted in pulmonary dysfunction and morphological abnormalities, and these CS-induced phenotypes were mitigated in mice in which *Arf*-expressing cells were eliminated prior to CS exposure. We also showed that the elimination of *Arf*-expressing cells following lung injury was beneficial in cigarette smoke extract (CSE)-administered mice. Collectively, these results imply that senolysis has potential as a therapeutic approach for human emphysema.

## 2. Materials and Methods

### 2.1. Animals

All animal experiments were approved by and conducted in accordance with guidelines established by the National Center for Geriatrics and Gerontology Animal Ethics Committee (approval numbers: 28-6, 29-24, 30-34, and 31-3). Female ARF-DTR mice [19] and littermate mice (wild-type) with the C57BL/6J (originally obtained from Japan SLC, Inc., Shizuoka, Japan) background were maintained under specific pathogen-free conditions with a 12-h light/dark cycle, constant temperature and ad libitum access to food (CE-2; CLEA Japan, Inc., Tokyo, Japan) and water. DT was intraperitoneally injected at a concentration of 50 μg/kg body weight. Twenty-five each of ARF-DTR and wild-type mice were used for the experiments including preliminary experiments and data/sample acquisition. Body weight and condition were monitored every week to assess the animal health during the experiment. All reasonable efforts were made to minimize animal pain and suffering.

### 2.2. CS and CSE Treatment

CS was prepared using commercially marketed Peace non-filter cigarettes (Japan Tobacco, Tokyo, Japan) containing 29 mg of tar and 2.5 mg of nicotine per cigarette. CS exposure was performed on a tobacco smoke inhalation experiment system for small animals (Sibata Scientific Technology Ltd, Saitama, Japan) [22,23]. CS was diluted with compressed air to 3.5% and exposed to mice at a rate of 6 puffs per min with a 15-mL stroke volume. Mass concentration of diluted CS was 1.6–1.8 mg/m^3^. Mice were exposed to diluted CS or fresh air (control group) for 30 min per day, 5 days per week for 4 weeks.

CSE was prepared according to previously described methods [24,25]. Smoke from 8 Peace non-filter cigarettes was drawn through 100-mL sterile phosphate-buffered saline (PBS) using a peristaltic pump at a rate of 1.0 mL per min. Each cigarette was burned for approximately 6 min. CSE was cleared by centrifugation and 50 μL was intranasally administered to mice. Mice were anesthetized with 2% isoflurane prior to the CSE administration (Wako Pure Chemicals Industries, Ltd., Osaka, Japan). CSE was freshly prepared prior to each set of experiments.

### 2.3. In Vivo Imaging Analysis

An in vivo luciferase imaging analysis was performed using the IVIS imaging system (Perkin Elmer, Waltham, MA, USA). Mice were anesthetized with 2% isoflurane prior to the luciferin injection (150 mg/kg body weight, VivoGlo; Promega, Madison, WI, USA). Luciferase bioluminescence was monitored 10 min after the luciferin injection.

### 2.4. Morphometric Analysis

Morphometric analysis was performed as previously described [20]. Lung tissues were fixed with Mildform^®^20N (Wako Pure Chemicals Industries, Ltd., Osaka, Japan) at constant pressure (25 cm H_2_O). Paraffin-embedded section (5 μm thickness) was stained with hematoxylin and eosin. All histopathological analyses were performed in a blinded manner.

### 2.5. Immunofluorescence

Paraffin-embedded lung sections were deparaffinized, washed with PBS and antigen retrieval was performed by autoclave (120 °C for 20 min) in 10 mM citric acid buffer (pH 9.0) or by L.A.B. solution (Polysciences, Inc., Warrington, PA, USA). The sections were blocked with 10% donkey serum (ImmunoBioScience, Mukilteo, WA, USA) for 1 h at room temperature. The section were immunostained with rabbit polyclonal antibody against p19^Arf^ (1:200 dilution, cat# ab80, Abcam, Cambridge, UK), rat monoclonal antibody against p19^Arf^ (1:100 dilution, cat# sc-32748, Santa Cruz Biotechnology, Dallas, TX, USA), rabbit polyclonal antibody against p16^Ink4a^ (1:100 dilution, cat# sc-1207, Santa Cruz Biotechnology, Dallas, TX, USA) and rat monoclonal antibody against EpCAM (1:200 dilution, cat# 552370, BD Biosciences, San Jose, CA, USA) overnight at 4 °C. After three washes in PBS, the sites of antibody binding were detected using Alexa Fluor® 488-labeled donkey anti-rat IgG (Jackson ImmunoResearch Labs, cat# 712-545-153, West Grove, PA, USA), Alexa Fluor® 647-labeled donkey anti-rabbit IgG (Jackson ImmunoResearch Labs, cat# 711-606-152, West Grove, PA, USA), Alexa Fluor^®^ 647-labeled donkey anti-rat IgG (Jackson ImmunoResearch Labs, cat# 712-606-150, West Grove, PA, USA) and Alexa Fluor® 488-labeled donkey anti-rabbit IgG (Jackson ImmunoResearch Labs, cat# 711-545-152, West Grove, PA, USA). Sections were mounted in Prolong Gold antifade reagent with DAPI (Life Technologies, Carlsbad, CA, USA). Immunofluorescence images were taken using an inverted microscope (Biorezo BZ-9000, Keyence, Osaka, Japan). Images were acquired using BZ-II Viewer software (Keyence, Osaka, Japan). For the quantitation of p19^Arf^-positive cells, multiple fields containing more than a hundred cells were analyzed in each sample.

### 2.6. RNA Analysis

A real-time PCR analysis was performed as previously described [26]. Total RNA was isolated from lung tissues using the PureLink^®^ RNA Mini kit (Thermo Fischer Scientific, Waltham, MA, USA), and reverse-transcribed using the PrimeScript RT reagent kit with a gDNA eraser (TAKARA BIO, Shiga, Japan) according to the manufacturer’s instructions. PCR was performed on a CFX Real Time System (BioRad, Hercules, CA, USA) using the KOD SYBR qPCR mix (TOYOBO Co., Ltd., Osaka, Japan). The following primers were utilized for the amplification of specific genes: *Arf*; sense 5′-GCCGCACCGGAATCCT-3′ and antisense 5′-TTGAGCAGAAGAGCTGCTACGT-3′, *Ink4a*; sense 5′-CCCAACGCCCCGAACT-3′ and antisense 5′-GCAGAAGAGCTGCTACGTGAA-3′, *Mmp-12*; 5′-CTGCTCCCATGAATGACAGTG-3′ and antisense 5′-AGTTGCTTCTAGCCCAAAGAAC-3′, *Elane*; sense 5′-CCTTGGCAGACTATCCAGCC-3′ and antisense 5′-GACATGACGAAGTTCCTGGCA-3′, *DTR-Luc* (fusion); sense 5′-TTTAGGTACCATAGGAGAGGAGG-3′ and antisense 5′-CATCTTCCAGCGGATAGAATGGC-3′, *Gapdh*; 5′-AATGGTGAAGGTCGGTGTG-3′ and antisense 5′-GAAGATGGTGATGGGCTTCC-3′.

### 2.7. Pulmonary Function Test

Pulmonary function tests were performed on a FlexiVent system (SCIREQ, Montreal, Quebec, Canada) as previously described [19,27]. Mice were euthanized by an intraperitoneal injection of excess amount of pentobarbital sodium (100 mg/kg of body weight), and connected to the FlexiVent system after tracheotomy. Diaphragm was removed prior to the spirometry analyses.

### 2.8. Bronchoalveolar Lavage Fluid (BALF) Analysis

BALF cells were analyzed as previously reported [28]. Briefly, cells collected from BALF were attached to slides using StatSpin Cytofuge (Beckman Coulter, Brea, CA, USA), and subjected to modified Giemsa staining using the Diff-Quick stain kit (Sysmex, Kobe, Japan).

## 3. Results

### 3.1. Cigarette Smoking Induces Arf and Ink4a Expression in Mice

**Figure 1:**
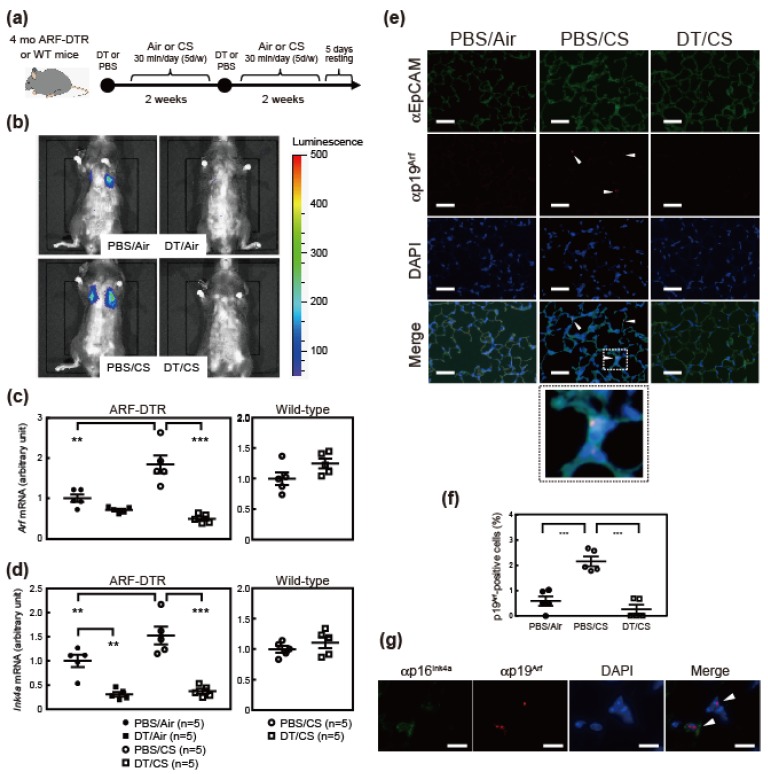
Cigarette smoke (CS) increased *Arf/Ink4a* levels in ARF-DTR mice. (**a**) Experimental schedule. Four-month-old ARF-DTR mice were exposed to CS for 4 weeks. Diphtheria toxin (DT) or phosphate-buffered saline (PBS) was intraperitoneally administered twice with a 2-week interval. (**b**) An in vivo imaging analysis was performed 4 weeks after the CS exposure. Representative images are shown. (**c**,**d**) The expression of (**c**) *Arf* and (**d**) *Ink4a* mRNA was analyzed by real-time PCR in the lung tissue of ARF-DTR or wild-type mice. Data were normalized to *Gapdh* in each group. (**e**) Representative images of immunostained lung sections of control (Air), CS and DT/CS ARF-DTR mice using epithelial cell marker EpCAM (green) and ARF-DTR mice using p19^A^^rf^ (red) antibodies. Sections were counterstained with DAPI (blue). Bar; 40 μm. Arrowheads indicate p19^Arf^-expressing cells. Dotted line (CS, merge) indicates magnified area (CS, bottom). (**f**) The number of p19^Arf^-positive epithelial cells in lung tissues of control, CS, DT/CS ARF-DTR mice was counted. (**g**) Representative images of CS lung section immunostained using p16^Ink4a^ (green) and p19^Arf^ (red) antibodies. Section was counterstained with DAPI. Arrowheads indicate p19^Arf^ and p16^Ink4a^-expressing cells. Bar; 10 μm. In (**c**), (**d**) and (**f**), bars represent means ± SEM. Data were analyzed by a one-way ANOVA and Tukey post-hoc analysis. Student’s *t*-test was performed for the analysis of wild-type mice data and no significance was observed. ** *p* < 0.01 and *** *p* < 0.001.

To investigate the roles of p19^Arf^-expressing cells in the CS-induced lung pathology, 4-month-old ARF-DTR or wild-type mice were exposed to CS as previously described [29] for 4 weeks (30 min per day, 5 days per week), as shown in Figure 1a. DT was administered twice with a 2-week interval, and initial administration was performed prior to the CS exposure. We initially performed an in vivo imaging analysis to monitor lung luciferase expression, which was controlled by *Arf* promoter/enhancer activity in ARF-DTR mice [19]. Luciferase activity in the chest region, which represents the expression of luciferase in lung tissue [19], was slightly increased in CS-exposed ARF-DTR mice (Figure 1b). RNA was then extracted from lung tissue and the expression of *Arf* and *Ink4a* mRNA was analyzed. CS exposure resulted in an increase in both *Arf* and *Ink4a* mRNA levels (Figure 1c,d), which is in contrast with previous findings obtained from an elastase-induced emphysema model showing no significant change in the mRNA levels of these genes [20]. Consistent with their roles in cellular senescence, the number of cells stained with Sudan Black B (SBB) was increased in CS-exposed lungs, suggesting that cellular senescence is enhanced in these mice (Supplementary Figure S1) [30]. Additionally, p19^Arf^, which is generally undetectable in normal tissues but is expressed in a low percentage of mesenchymal cells in the lung of adult or elastase-treated animals [19,20], became apparent in a low number of epithelial cells after CS exposure (Figure 1e,f), suggesting that chronic insult by CS could damage the epithelial cells. *Arf* mRNA was barely detectable in BALF cell of which major component is macrophage (Supplementary Figure S2).

The administration of DT resulted in significant reductions in *Arf* and *Ink4a* mRNA levels (Figure 1c,d). The number of p19^Arf^-expressing cells was also decreased by DT treatment (Figure 1e,f). The down-regulation of *Ink4a* in DT-treated lungs likely reflects the co-expression of p19^Arf^ and p16^Ink4a^ in lung (Figure 1g). Consistently, luciferase activities were under the detection limit after the DT treatment (Figure 1b). SBB staining suggested that senescent cells were eliminated by DT-treatment in CS-exposed animals (Supplementary Figure S1). On the other hand, DT had no effect on *Arf* or *Ink4a* mRNA levels in wild-type mice, which confirmed that the expression of these genes was ablated through the transgene (Figure 1c,d).

### 3.2. Ablation of Arf Expression Attenuates CS-Induced Lung Pathologies

**Figure 2:**
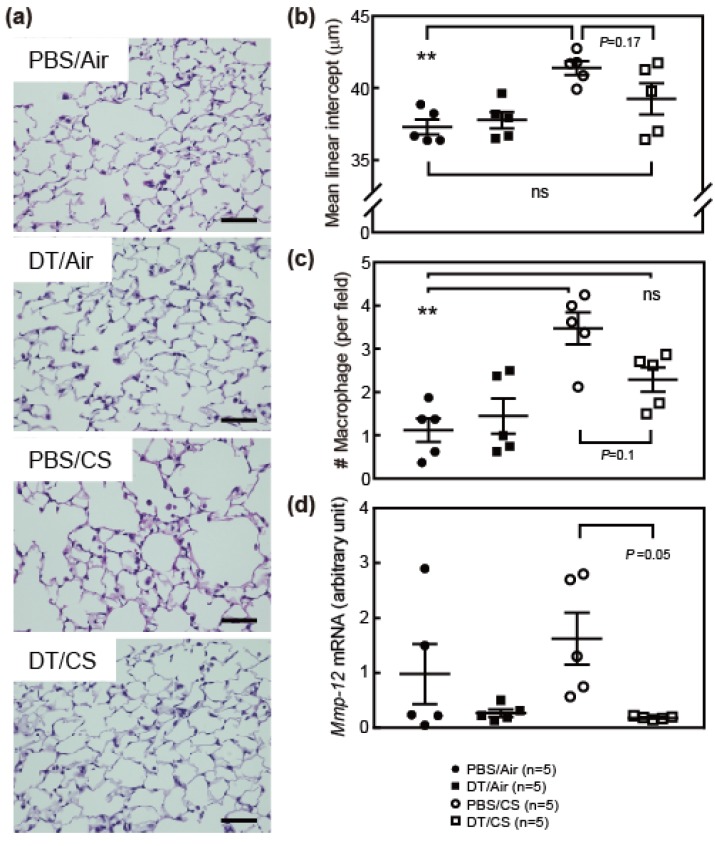
Ablation of *Arf*-expressing cells ameliorated CS-induced lung pathologies. (**a**) Representative images of ARF-DTR lung sections stained with hematoxylin and eosin. Bar; 50 μm. (**b**) Alveolar mean linear intercepts were measured. (**c**) The number of macrophages per field (×40) was counted. At least 8 fields were analyzed in each mouse. (**d**) The expression of *Mmp-12* was analyzed by real-time PCR. Data were normalized to *Gapdh* in each group. Bars represent means ± SEM. Data were analyzed by a one-way ANOVA and Tukey post-hoc analysis. ** *p* < 0.01 and ns; not significant.

We analyzed the effects of CS and DT in ARF-DTR mouse lung tissue. After a 4-week exposure to CS, lung tissues were inflated with fixative solution at a constant pressure prior to sectioning. As shown in Figure 2a, partial alveolar wall destruction was observed in the CS-exposed (PBS/CS) ARF-DTR mouse lung, which appeared to be suppressed in DT-treated ARF-DTR mice. To obtain more quantitative data, we evaluated the alveolar size by measuring the mean linear intercept (chord) length. DT did not affect the alveolar size at this age, while it was reduced by DT in the older animals [19] (Figure 2b). CS exposure resulted in a significant increase in the alveolar size, but no significance was observed in the presence of DT treatment, suggesting that the elimination of *Arf*-expressing cells protected the tissue from CS-induced alveolar collapse. We previously suggested that the elimination of p19^Arf^-expressing cells suppressed elastase-induced alveolar inflammation, thereby protecting tissue from emphysema-associated pathologies [20]. This may also be the case in the CS model because the number of macrophages, which was increased in CS-exposed mice, was maintained in DT-treated animals (Figure 2c). *Matrix metalloproteinase* (*Mmp*)*-12* was consistently down-regulated in lung tissue after the DT treatment (Figure 2d). DT did not markedly affect alveolar sizes, macrophage numbers or *Mmp-12* expression in CS-exposed wild-type mice (Supplementary Figure S3). Collectively, these results suggest that similar to the elastase-induced lung injury model, the CS-induced lung pathology was attenuated by the elimination of p19^Arf^-expressing cells.

While macrophages express *Ink4a* (Supplementary Figure S2) and senescence-associated β-galactosidase [31], *Arf* and *DTR* mRNA levels were barely detectable in BALF cells and unchanged by DT, excluding the possibility that DT directly exerts cytotoxicity in macrophages.

### 3.3. Arf-Expressing Cells Facilitates CS-Induced Pulmonary Dysfunction

**Figure 3:**
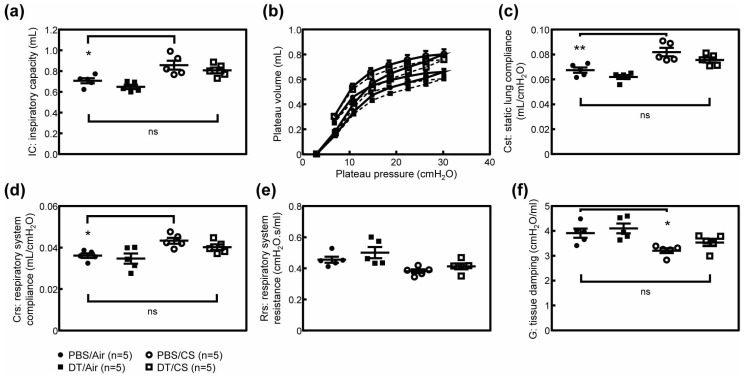
Pulmonary function was maintained in the absence of *Arf*-expressing cells after CS exposure. (**a**) Inspiratory capacity was measured in each mouse. (**b**) Pressure-volume curves of ARF-DTR mice lungs. (**c**) Static lung compliance. (**d**,**e**) Respiratory system compliance and resistance were obtained using a single frequency forced oscillation technique. (**f**) Tissue damping coefficient. Data represent means ± SEM. Data were analyzed by a one-way ANOVA and Tukey post-hoc analysis. * *p* < 0.05, ** *p* < 0.01 and ns; not significant.

The destruction of alveolar walls by CS or elastase leads to a decline in pulmonary function, which is exemplified by increased lung tissue compliance. Hence, we tested whether the elimination of p19^Arf^-expressing cells restores pulmonary function in CS-exposed ARF-DTR mice. Inspiratory capacity (IC) was significantly increased in CS-exposed mice (Figure 3a). Consistently, lung tissue compliance (Cst; static lung compliance, Crs; respiratory system compliance) was significantly increased in CS-exposed mice (Figure 3b–d), while respiratory system resistance (Rrs) was unchanged (Figure 3e). However, the effects of CS were diminished or prevented when DT was administered prior to the CS exposure in ARF-DTR (Figure 3 and Supplementary Figure S4). Tissue damping (G) that reflects the viscoelastic properties of lung tissue was decreased in CS-exposed mice (Figure 3f). These changes were consistent with the phenotypes observed in *Timp-3* knockout, *SP-D* knockout or *Notch* mutant mice that spontaneously develop pulmonary emphysema [32,33,34]. DT partly suppressed the CS-induced change in tissue damping coefficient in ARF-DTR (Figure 3f and Supplementary Figure S4). Collectively, these results suggest that the presence of *Arf*-expressing cells exacerbates CS-induced lung dysfunction through enhancements in the recruitment of macrophages, and that elimination of *Arf*-expressing cells leads to the re-establishment of respiratory function or protection it from CS-induced damage.

### 3.4. Effects of p19^Arf^-Expressing Cell Ablation in Cigarette Smoke Extract-Treated Mice

**Figure 4:**
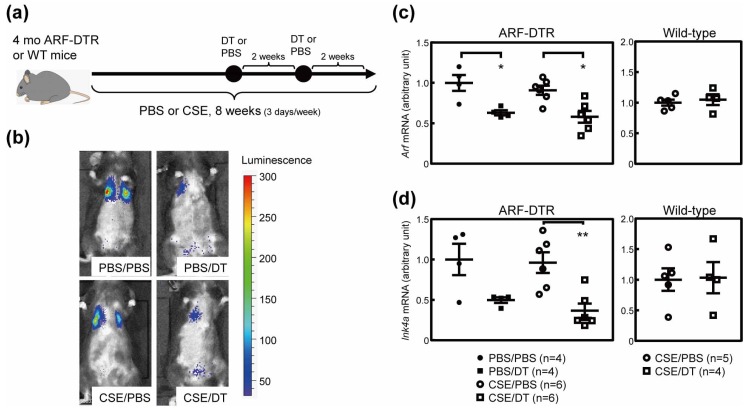
Cigarette smoke extract (CSE) inhalation model. (**a**) Experimental schedule. CSE or PBS was intranasally administered 3 times per week for 8 weeks to 4-month-old ARF-DTR or wild-type mice. DT was intraperitoneally injected twice with a 2-week interval. (**b**) Representative images of an in vivo imaging analysis of luciferase expression. (**c**,**d**) The expression of (**c**) *Arf* and (**d**) *Ink4a* mRNA was analyzed by real-time PCR in the lung tissue of ARF-DTR or wild-type mice. Data were normalized to *Gapdh* in each group. Bars represent means ± SEM. Data were analyzed by a one-way ANOVA and Tukey post-hoc analysis. Student’s *t*-test was performed for the analysis of wild-type mice data and no significance was observed. * *p* < 0.05 and ** *p* < 0.01.

The present results and previous findings suggest that the elimination of p19^Arf^-expressing cells prior to the CS exposure or elastase inhalation exerts protective effects against emphysema-associated lung pathologies [20]. However, it currently remains unclear whether the elimination of p19^Arf^-expressing cells exerts therapeutic effects after lung injury. To test this possibility, we treated mice with DT after a damage-causing stimulation in ARF-DTR mice. In this experiment, we employed CSE inhalation as a lung injury model. CSE contains fewer chemical components than CS [35], but is sufficient to induce pulmonary inflammation [36]. Additionally, CSE has been shown to induce cellular senescence in cultured cells [37]. CSE was intranasally administered to ARF-DTR or wild-type mice for 8 weeks (3 times per week), as shown in Figure 4a. In this experiment, DT was administered 4 weeks after the CSE stimulation.

In contrast to the CS model (Figure 1), no marked differences were observed in lung luciferase activity between PBS- and CSE-treated animals (Figure 4b). Real-time PCR analyses consistently revealed that *Arf* and *Ink4a* expression remained unchanged in CSE-treated lung tissue (Figure 4c,d). Nevertheless, the DT treatment significantly reduced luciferase activity as well as *Arf* and *Ink4a* expression in both control and CSE-treated animals, but had no effect on the expression of these genes in wild-type mice (Figure 4b–d).

### 3.5. CSE Induced Pulmonary Dysfunction in Mice

**Figure 5:**
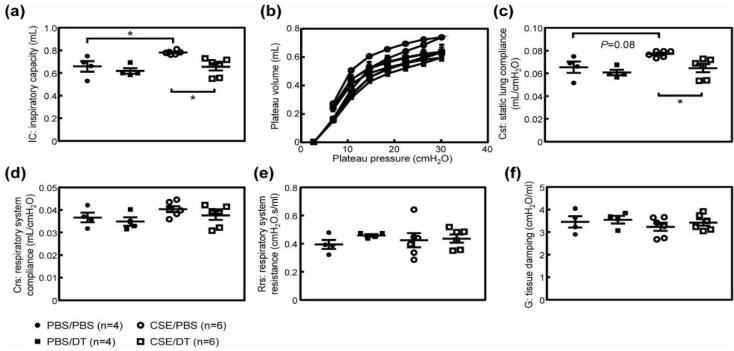
Effects of CSE on pulmonary function in ARF-DTR mice. (**a**) Inspiratory capacity, (**b**) pressure-volume loop, (**c**) static lung compliance, (**d**) respiratory system compliance, (**e**) respiratory system resistance, (**f**) tissue damping in ARF-DTR mice were shown. Data represent means ± SEM. Data were analyzed by a one-way ANOVA and Tukey post-hoc analysis. * *p* < 0.05.

Similar to the CS exposure, the CSE treatment also leads to impaired pulmonary function [38,39]. We performed pulmonary function tests in CSE-treated mice. The CSE treatment resulted in increases in IC and Cst (Figure 5a–c), although its effect may be much weaker than that of CS. Morphometric analysis also suggest that CSE had weaker effect, as no significant change was observed in alveolar size in CSE-treated animals (Supplementary Figure S5). Other parameters including Crs, Rrs and G were unaffected by the CSE treatment (Figure 5d–f), suggesting that CSE exerts differential effects on pulmonary function or is less effective than in the CS-exposure model. IC and Cst were both restored in the DT-treated group (Figure 5a–c), while DT had no effect on these parameters in CSE-treated wild-type animals (Supplementary Figure S6).

**Figure 6:**
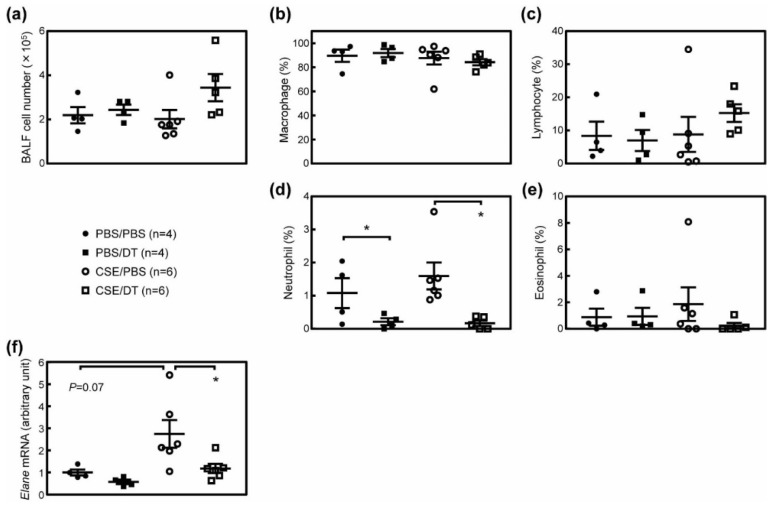
Effects of p19^Arf^ ablation on the BALF cell population. (**a**) The numbers of cells recovered from the BALF of ARF-DTR mice are shown. (**b**–**e**) The populations of (**b**) macrophages, (**c**) neutrophils, (**d**) eosinophils and (**e**) lymphocytes in BALF cells are shown. (**f**) The expression of neutrophil elastase (*Elane*) was analyzed by real-time PCR. Data were normalized to *Gapdh* in each sample. Data represent means ± SEM. Data were analyzed by a one-way ANOVA and Tukey post-hoc analysis. * *p* < 0.05.

We then analyzed cells in the BALF of these mice. While we and others have previously shown that the total cell number in BALF was significantly increased in the elastase-induced lung injury model [20,28,40], no consistent change was observed in the number of BALF cells (Figure 6a). This was somewhat expected because the continuous inhalation of PBS was sufficient to increase BALF cells [24]. Consistent with this observation, the number of macrophages, which accounts for more than 80% of the total BALF cell number, was unchanged by the CSE or DT treatment (Figure 6b). Other cells, including neutrophils, lymphocytes and eosinophils, were also unaffected by the CSE treatment (Figure 6c–e). While the administration of DT reduced the macrophage number in CS-treated ARF-DTR mice (Figure 2) and the elastase-induced emphysema model [20], it had no effect in CSE-treated ARF-DTR mice (Figure 6b). Nevertheless, we observed a significant decrease in the neutrophil number in both PBS and CSE-treated ARF-DTR mice by DT (Figure 6d), but not in CSE-treated wild-type mice (Supplementary Figure S7). Consistently, neutrophil elastase (*Elane*) levels were also decreased by DT treatment in ARF-DTR mice (Figure 6f). Collectively, these results suggest that CS and CSE differentially induce lung pathologies, while both stimulations lead to pulmonary dysfunction exemplified by increased lung tissue compliance, and the ablation of *Arf*-expressing cells exerts beneficial effects, even after the damage-inducing stimulation.

## 4. Discussion

The present study demonstrated that the elimination of *Arf*-expressing cells ameliorated CS-induced lung pathologies using ARF-DTR mice. In contrast to INK-ATTAC and p16-3MR mice [41,42], ARF-DTR mice express the transgene (DTR and luciferase) under the control of the *Arf* promoter/enhancer [19]. While the role of *ARF* in human cellular senescence and aging remains controversial, *Arf* is essential for senescence in rodent cells [43]. Additionally, the DT treatment decreased *Ink4a* as well as *Arf* expression, indicating *Arf*- and *Ink4a*-positive senescent cells were eliminated from the lung tissue of ARF-DTR mice. The elimination of *Arf*-expressing cells prior to the CS inhalation trended toward protection against CS-induced alveolar enlargement, macrophage infiltration and pulmonary dysfunction. Additionally, the present results obtained with the CSE inhalation model suggest that the elimination of *Arf*-expressing cells after the stimulation still exerted beneficial effects on lung function.

The etiology of COPD is complex because many factors contribute to the development of this disease [1,2] and its biology remains poorly understood. While cigarette smoking is the predominant risk factor for COPD, a relatively small proportion of smokers develop the disease. This underscores other factors in addition to CS influencing susceptibility to the disease. The incidence of COPD is increased by aging irrespective of the smoking history [44], which highlights aging-associated changes in pulmonary and/or other organs facilitating the development of this disease. Cellular senescence may be a key factor that promotes disease-associated pathologies. Cellular senescence is accelerated in emphysema patients [7], and we previously suggested that cellular senescence plays a role in CS-induced lung pathologies [29], while aging-associated changes other than cellular senescence may also contribute to the CS-induced pathology [45].

We previously reported that the elimination of *Arf*-expressing cells protected against elastase-induced emphysema [20]. Elastase inhalation is a facile and highly reproducible model for the induction of pulmonary emphysema, and a single shot of elastase is sufficient to induce alveolar collapse. However, the elastase treatment results in panlobular (panacinar) emphysema, which is a rare disease in humans. In humans, centrilobular (centriacinar) emphysema is the most common type of pulmonary emphysema, the main cause of which is smoking. Therefore, we investigated the effects of senolysis in the CS mouse model in the present study. In contrast to the elastase model, CS exposure resulted in weak, but chronic damage to the lung tissue, which leads to impaired pulmonary function. We also employed CSE inhalation for the lung injury model. CSE contains fewer chemical components than CS [35]. However, in contrast to the CS model in which the whole body is exposed to CS, the CSE inhalation model may more specifically target the tissue/organ. The present results suggest that CSE inhalation was sufficient to induce pulmonary dysfunction; however, it did not induce notable histological changes as previously documented [36]. In both models, the elimination of *Arf*-expressing cells was proven to ameliorate lung damage, suggesting that senescent cells also play an important role in CS-induced pathologies.

Inflammation in lung tissue is a critical step for the development of disease. The inactivation of macrophage or neutrophil elastase has been shown to confer resistance to CS-induced emphysema in mice [5,6]. The results obtained herein indicate that CS and CSE differentially affect alveolar inflammatory cells. Nevertheless, the elimination of *Arf*-expressing cells suppressed the expression of elastolytic enzymes, suggesting that senescent cells exacerbate CS/CSE-induced lung pathologies by enhancing alveolar inflammation. Hall and colleagues recently reported that p16^Ink4a^ is expressed in non-senescent macrophages [31,46]. These findings suggested that the DT treatment ablated non-senescent macrophages, thereby affecting pulmonary inflammation and the associated phenotypes. However, this is unlikely because endogenous *Arf* levels are extremely low and the expression of the transgene (DTR and luciferase) was not detected in the macrophages of ARF-DTR mice (Supplementary Figure S2) [19]. Furthermore, our previous findings indicated that p19^Arf^ was present in the mesenchymal cells of lung tissue; however, the population of *Arf*-expressing cells was estimated to be as small as 2% of all mesenchymal cells in lung tissue. Senescent cells produce chemokines/cytokines as part of SASP, which have a marked impact on the inflammatory process [47]. Hence, it is reasonable to assume that the effects of DT were attributed to the clearance of senescent cells that may facilitate the inflammatory response through SASP.

We previously demonstrated that the elimination of *Arf*-expressing cells exerted preventive effects against elastase-induced pathologies [20]. The present results obtained with the CSE model strongly suggest that senolysis also exhibits therapeutic efficacy because DT partly restored pulmonary function in ARF-DTR mice in which lung injury was induced by CSE in advance. Thus, these results suggest the potential of the application of senolytic drugs as emphysema therapy. In this regard, it is important to note that the oral administration of ABT-263/Navitoclax exerted beneficial effects against elastase-induced emphysema [20]. While ABT-263/Navitoclax has side effects [48,49] and may not be directly applied to senolysis in humans, the targeting of senescent cells will be an effective approach. Further studies are required to assess the therapeutic efficacy of senolysis and senolytic drugs for the treatment of emphysema.

## 5. Conclusions

Elimination of p19^Arf^-expressing cells ameliorated the CS- and CSE-induced pulmonary dysfunction in mice. These findings suggest emphysema-associated pathologies are exacerbated by cellular senescence, and that targeting senescent cells may be an effective approach for COPD therapy.

## Supplementary Materials

The following are available online at https://www.mdpi.com/2218-273X/10/3/462/s1, Figure S1: Effects of CS and DT on Sudan Black B staining, Figure S2: Expression of *Ink4a*, *Arf* and *DTR/Luc* mRNA in BALF cells, Figure S3: DT had no effect on alveolar size, macrophage numbers, or *Mmp-12* expression in wild-time mice, Figure S4: DT had no effects on the pulmonary function of CS-exposed wild-type mice, Figure S5: CSE had no effect on lung morphology in ARF-DTR mice, Figure S6: DT had no effect on the pulmonary function of CSE-treated wild-type mice, Figure S7: DT had no effects on the BALF cell population in CSE-treated wild-type animals.
