# Supplementary material for: p19Arf Exacerbates Cigarette Smoke-Induced Pulmonary Dysfunction
Source: Biomolecules. 2020 Mar 17;10(3):462. doi: 10.3390/biom10030462 (PMC7175375; doi:10.3390/biom10030462)
Supplement: Supplementary file 1 [file biomolecules-10-00462-s001.pdf]

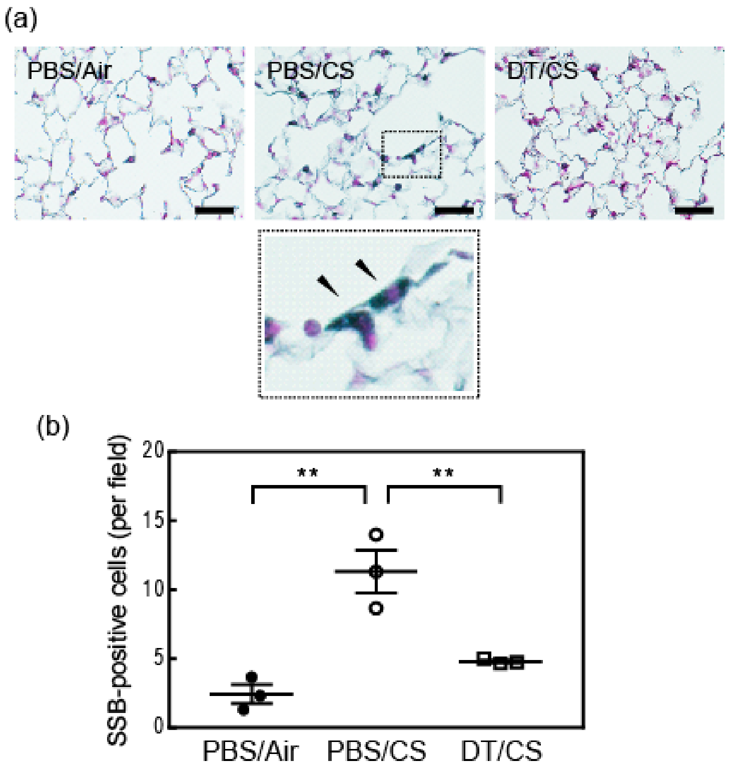

Figure S1. Effects of CS and DT on Sudan Black B staining. (a) Representative images of SBB staining. Sections were counterstained by Nuclear Fast Red. Bar; 40 μm. Dotted line indicates magnified area in PBS/CS. Arrowheads in the magnified image indicate cells stained by SBB. (b) SBB-positive cells per field were counted. Bars represent means ± SEM. Data were analyzed by a one-way ANOVA and Tukey post-hoc analysis. ***P*<0.01.

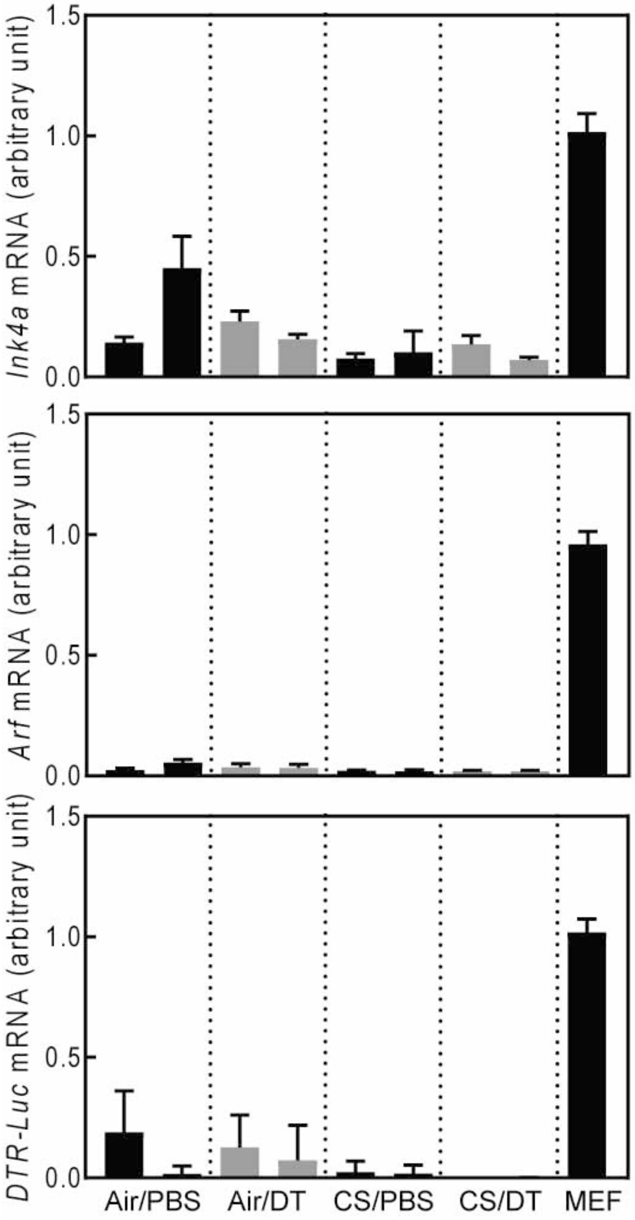

Figure S2. Expression of *Ink4a*, *Arf* and *DTR/Luc* mRNA in BALF cells. BALF cells were collected form ARF-DTR mice exposed to CS or control air with or without DT treatment (Fig1A). Two independent mice were analyzed in each group. Embryonic fibroblasts of ARF-DTR mice (MEF) were used for the comparison. Data represents the means ± SD of the triplicate samples.

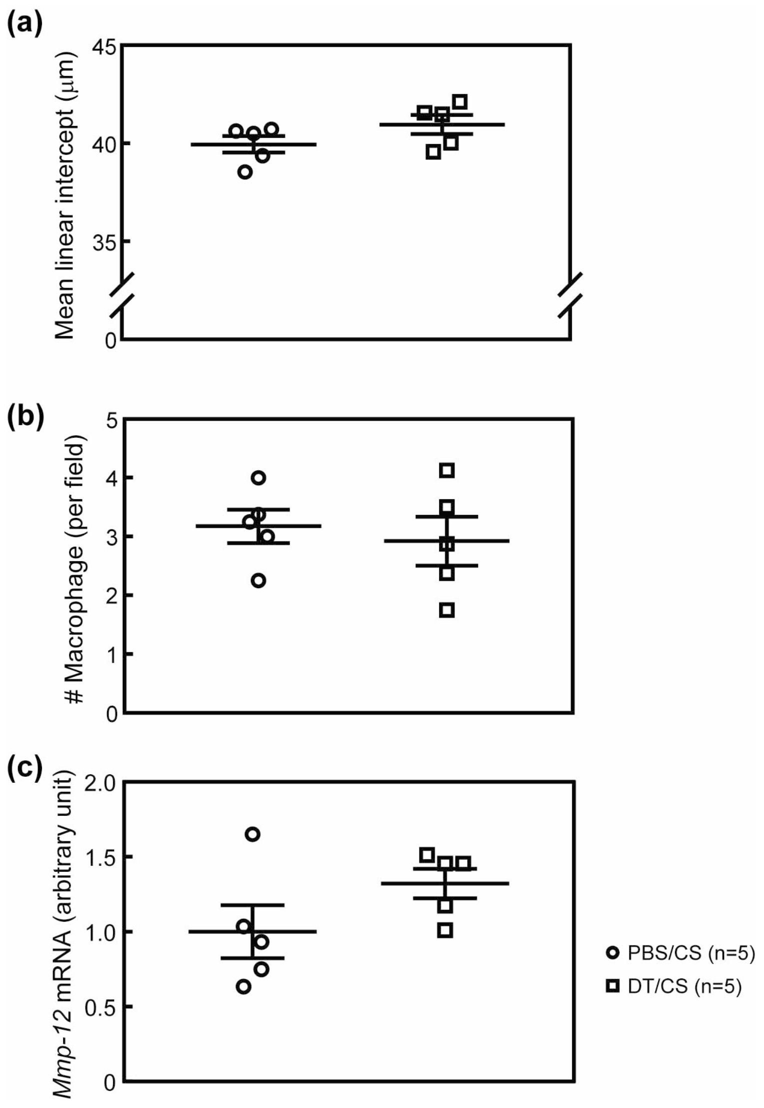

Figure S3. DT had no effect on alveolar size, macrophage numbers, or *Mmp-12* expression in wild-time mice. (**a** and **b**) Alveolar mean linear intercepts and the number of macrophages per field (×40) were measured. (**c**) The expression of *Mmp-12* was analyzed by real-time PCR. Data were normalized to *Gapdh* in each group. Data represent means ± SEM. Data were analyzed by the Student's *t*-test and no significance was observed.

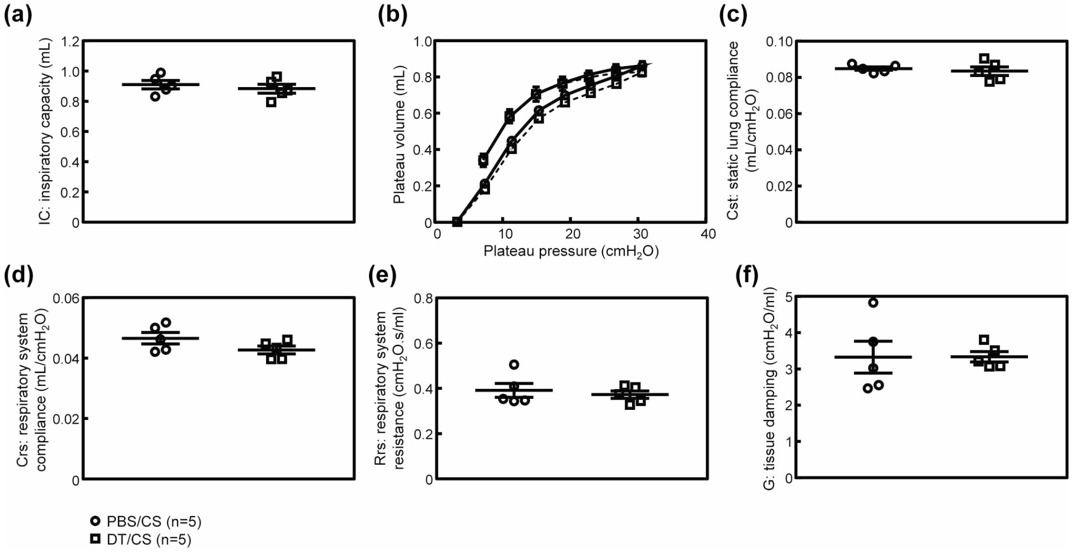

Figure S4. DT had no effects on the pulmonary function of CS-exposed wild-type mice. Inspiratory capacity (**a**), pressure-volume loop (**b**), static lung compliance (**c**), respiratory system compliance (**d**), respiratory system resistance (**e**), tissue damping (**f**) in ARF-DTR mice were shown. Data represent means ± SEM. Data were analyzed by a one-way ANOVA and Tukey post-hoc analysis. *P<0.05. Data represent means ± SEM. Data were analyzed by the Student's t-test and no significance was observed.

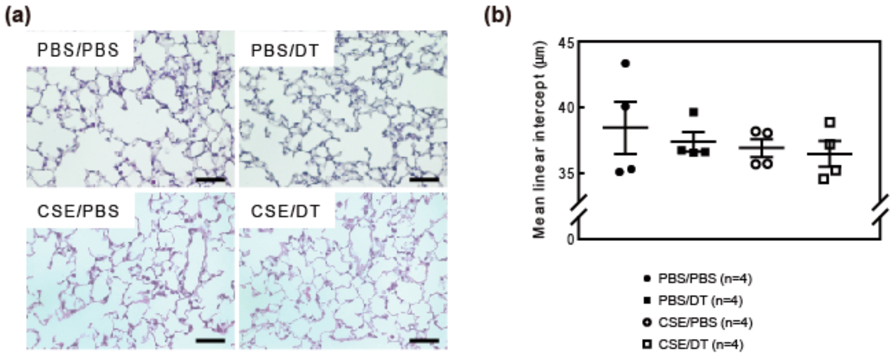

Figure S5. CSE had no effect on lung morphology in ARF-DTR mice. (**a**) Representative images of ARF-DTR lung sections stained with hematoxylin and eosin. Bar; 50 μm. (**b**) Alveolar mean linear intercepts were measured. No significance was detected by a one-way ANOVA.

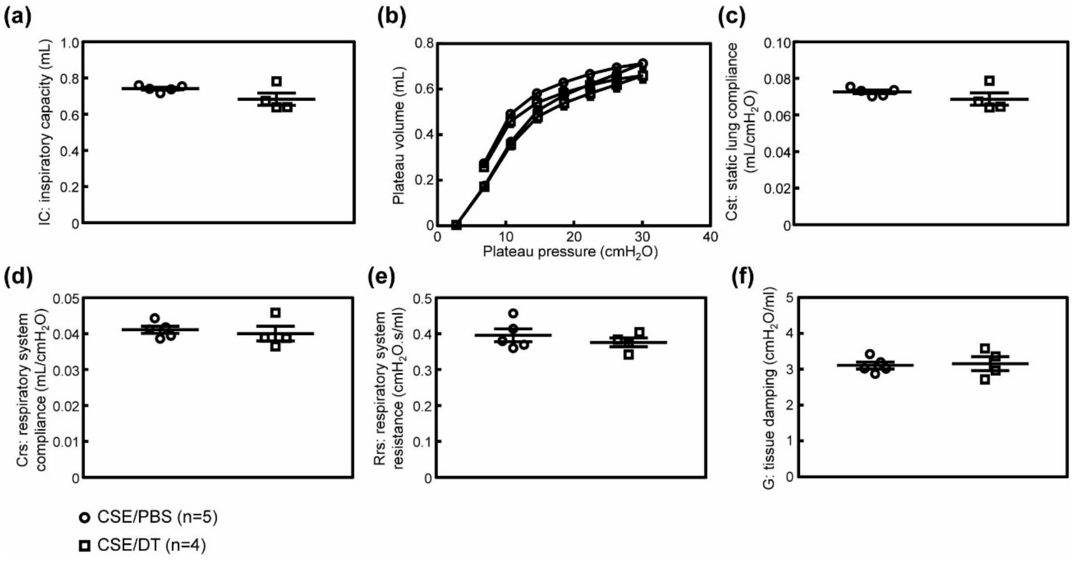

Figure S6. DT had no effect on the pulmonary function of CSE-treated wild-type mice. Inspiratory capacity (**a**), pressure-volume loop (**b**), static lung compliance (**c**), respiratory system compliance (**d**), respiratory system resistance (**e**), tissue damping (**f**) in ARF-DTR mice were shown. Data represent means ± SEM. Data were analyzed by a one-way ANOVA and Tukey post-hoc analysis. **P*<0.05. Data represent means ± SEM. Data were analyzed by the Student's *t*-test and no significance was observed.

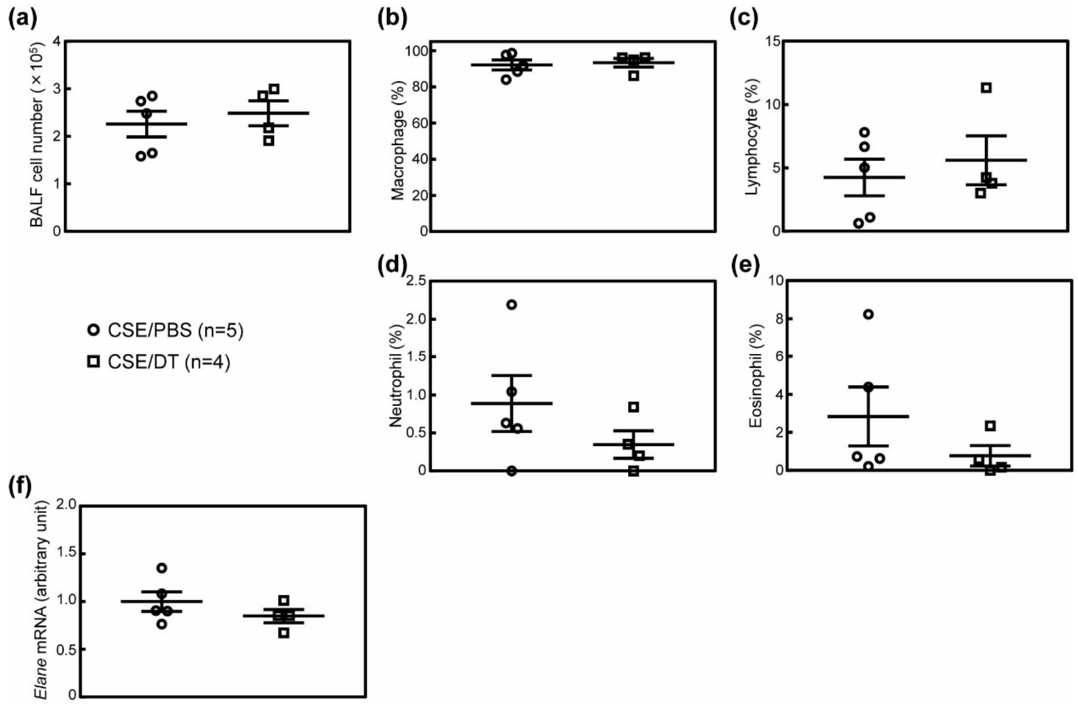

Figure S7. DT had no effects on the BALF cell population in CSE-treated wild-type animals. (**a**) The numbers of cells recovered from the BALF of wild-type mice are shown. (**b**-**e**) The populations of macrophages (b), neutrophils (c), eosinophils (d), and lymphocytes (e) in BALF cells are shown. (**f**) The expression of neutrophil elastase (*Elane*) was analyzed by real-time PCR. Data were normalized to *Gapdh* in each sample. Data represent means ± SEM. Data were analyzed by the Student's *t*-test and no significance was observed.
